# The Relationship Between the Adverse Events and Efficacy of Sorafenib in Patients With Metastatic Renal Cell Carcinoma: A Multicenter Retrospective Study from Northwest China

**DOI:** 10.1097/MD.0000000000002222

**Published:** 2015-12-11

**Authors:** Yu Zheng, Fuli Wang, Guojun Wu, Longlong Zhang, Yangmin Wang, Zhiping Wang, Peng Chen, Qing Wang, Jingyi Lu, Yujie Wang, Peijun Li, Jian Wang, Xitao Lu, Jianlin Yuan

**Affiliations:** From the Department of Urology, Xijing Hospital, Fourth Military Medical University, Xi’an, Shaanxi Province (YZ, FW, GW, LZ, JY); Department of Urology, General Hospital of Lanzhou Military Area Command of Chinese PLA (YW); Department of Urology, The Second Affiliated Hospital of Lanzhou University, Lanzhou, Gansu Province (ZW); Department of Urology, Affiliated Tumor Hospital of Xinjang Medical University (PC); Department of Urology, People's Hospital of Xinjiang Uygur Autonomous Region, Urumqi (QW); Department of Urology, Xinjiang karamay Central Hospital, Karamay (JL); Department of Urology, The First Affiliated Hospital of Xinjiang Medical University, Urumqi, Xinjiang Uyghur Autonomous Region (YW); Department of Urology, General Hospital of Ningxia Medical University, Yinchuan, Ningxia Hui Autonomous Region (PL); Department of Urology, Qinghai University Affiliated Hospital, Xining, QingHai Province (JW); and Department of Urology, First People's Hospital of Shizuishan, Shizuishan, Ningxia Hui Autonomous Region, China (XL).

## Abstract

The aim of the study is to evaluate the relationship between the adverse events and efficacy of sorafenib in patients with metastatic renal cell carcinoma (mRCC), with a purpose to guide the judgment of efficacy in sorafenib treatment.

Eighty-three mRCC patients who received sorafenib therapy at northwest China were studied retrospectively. Univariate and multivariate analyses were performed to correlate tumor response, progression-free survival (PFS), and overall survival (OS) with adverse event types and grades.

Among 83 patients who underwent sorafenib therapy, 2 cases (2.4%) had completed response (CR), 14 cases (16.9%) had partial response (PR), 57 cases (68.7%) had stable disease (SD), and 10 cases (12.0%) developed progressive disease (PD). The median PFS and OS were 15.0 and 29.0 months, respectively. The most frequent grade 1 or 2 adverse events included hand-foot syndrome (68.7%), diarrhea (54.2%), and alopecia (51.8%). The most common grade 3 or 4 adverse events were hand-foot syndrome (6.0%), hypertension (4.8%), and diarrhea (3.6%). The frequency and severity of adverse events correlated with tumor response rate (both with *P* < 0.05). Multivariate analysis showed the independent predictors of better PFS included rash (OR 0.307, 95%CI 0.148–0.636, *P* = 0.001) and diarrhea (OR 0.391, 95%CI 0.169–0.783, *P* = 0.008). Elevated transaminase was the independent predictor of poor PFS (OR 2.606, 95%CI 1.299–5.532, *P* = 0.012). For OS, rash (OR 0.473, 95%CI 0.253–0.886, *P* = 0.019) and diarrhea (OR 0.321, 95%CI 0.171–0.605, *P* = 0.000) correlated with better OS.

Sorafenib-related adverse events are associated with efficacy in patients with mRCC from northwest China. Rash and diarrhea are independent protective factors of both PFS and OS, and elevated transaminase is an independent risk factor of PFS. A large prospective study is warranted.

## Abstract 2

Supplemental Digital Content is available in the text

## INTRODUCTION

Renal cell carcinoma (RCC) is the most common malignant tumor of the kidney. Surgical resection is the best treatment for localized RCC. Approximately one-third of the patients have postoperative metastases or present with metastatic diseases.^1^ Targeted therapy has dramatically improved the treatment outcomes and the overall survival.

Sorafenib is one of the first targeted therapies approved for RCC based on clinical evidence of efficacy in several trials recently.^2–5^ It is an oral tyrosine kinase inhibitor (TKI) with small molecular weight. The most common TKI treatment-related adverse events are dermatological toxicity, gastrointestinal toxicity, hypertension, fatigue, serological change, and so on. In general, sorafenib is well tolerated with less adverse events.

Unfortunately, many patients do not benefit from the targeted treatment. A few studies have been performed to identify the influence factors and predict the efficacy of targeted therapies.^6^ We hypothesized that the therapeutic efficacy is dose-dependent. Thus, those patients who achieve high dose would have higher response rate than those with low-dose sorafenib. As the target tyrosine kinases of sorafenib also exist in normal cells, those patients with high-dose sorafenib will more likely develop more frequent and severer toxicity. Several studies have indicated that adverse events of targeted therapy are associated with the efficacy, such as nonsmall cell lung cancer (NSCLC),^7^ colorectal cancer,^8^ head and neck cancer.^9^ It is also suggested that the appearance of skin toxicity,^10^ hypertension,^11^ hypothyroidism^12^ correlated with improved tumor response rates and increased survival time of kidney cancer patients treated with targeted therapy.

Here we conducted a comprehensive retrospective review to determine the landscape of adverse events in metastatic RCC (mRCC) patients from northwest China who received sorafenib, and identified a few adverse events that correlated with tumor response, progression-free survival (PFS), and overall survival (OS).

## METHODS

### Patients and Treatment

This is a retrospective multicenter study. Data was collected from 279 patients at 10 medical centers in Northwest China since September 2006 to August 2014. All patients were pathologically diagnosed as mRCC. All patients had at least 1 measurable lesion according to the Response Evaluation Criteria In Solid Tumors (RECIST version 1.1).^13^ Among the 279 mRCC patients, 139 treated by sunitinib, 26 treated with cytokines, 12 patients changed TKIs into mammalian target of rapamycin (mTOR), and 6 with incomplete data were excluded. Among the remaining 96 patients who did not receive any systematic antitumor therapy before entering the group, 1 <18 years old, 12 with hepatic insufficiency (Child-Pugh C or above) or renal dysfunction (creatinine clearance < 30 mL/min) and a life expectancy of <12 weeks were also excluded. Thus, a total of 83 patients were included in the study which was supported by ethics committee of Xijing Hospital.

Sorafenib treatment was started 1 to 10 months after surgery or diagnosis, and lasted for at least 2 months to monitor drug-related adverse events. All patients orally received sorafenib 400 mg twice per day. If intolerable adverse events occurred, dose would be reduced to 400 mg daily until withdrawal. Patients would receive appropriate concomitant treatment for adverse events as well, but no antitumor therapy was conducted before disease progression. After treatment started, patients were followed up once a month for 3 months, then changed to once every 2 months until patients were taken off from the study. Tumor response was evaluated by imaging examinations (Computerized Tomography or magnetic resonance imaging) according to RECIST version 1.1.^13^

### Toxicity Assessment

Adverse events were recorded based on the types of adverse events, duration, and grades according to the Common Terminology Criteria For Adverse Events version 3.0 (CTCAE v3.0).^14^ Adverse events caused dose reduction or treatment discontinuation were also recorded.

### Statistical Analysis

Progression-free survival was defined as the time from the beginning of sorafenib treatment to tumor progression or death of patients. Overall survival was defined as the time that a patient was diagnosed with mRCC to death or last follow-up. The Kaplan–Meier method was used to evaluate PFS and OS, and the survival curves were plotted.

The chi square test was applied for the comparison of tumor response from different types and grades of adverse events. All the adverse events regarded as different variables and the Log-rank test was used for univariate analysis. Moreover, the multivariable Cox proportional hazards model was used to screen out adverse events associated with efficacy in mRCC by sorafenib therapy. All statistical significance (*P* < 0.05) were analyzed by SPSS 21.0.

## RESULTS

### Patient Characteristics and Efficacy

A total of 83 patients of mRCC was included in this study. The mean age was 55.08 ± 13.01 years old (range 19–81). The other characteristics of mRCC patients are shown in Table 1.

**TABLE 1:**
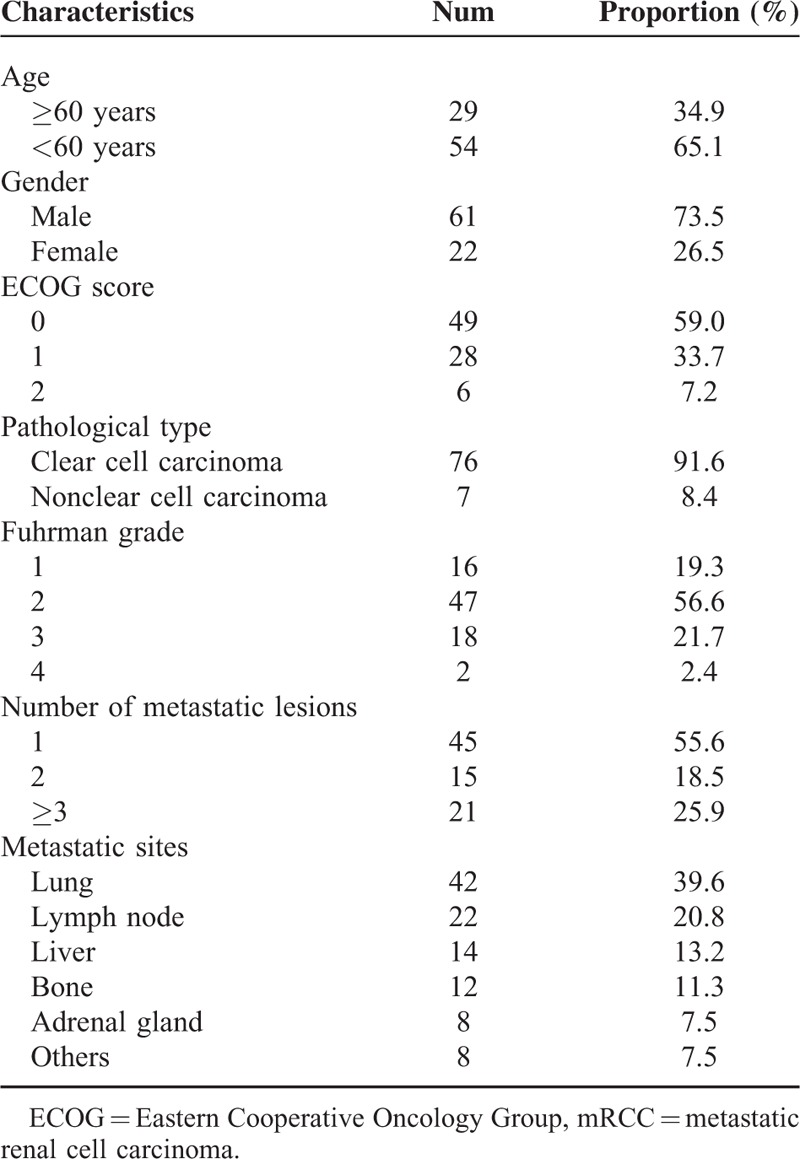
Basic Characteristics of mRCC Patients Treated With Sorafenib

Patients were followed up from 14 to 412 weeks, with a median follow-up of 56 weeks. By the time of data collection (at the end of August 2014), 8 patients were still alive, 1 patient died of cardiovascular disease and 5 patients lost follow-up. Radiologically confirmed completed response (CR), partial response (PR), stable disease (SD), and progressive disease (PD) were observed in 2 (2.4%), 14 (16.9%), 57 (68.7%), and 10 (12.0%) patients, respectively. The tumor control rate (CR + PR + SD) was 88.0% (73 / 83). The objective response rate (CR + PR) was 19.3% (16 / 83). The median PFS and OS were 15.0 and 29.0 months, respectively.

### Adverse Events

A total of 16 types of adverse events were recorded, and at least 1 adverse event occurred in each patient. The most frequent grade 1 or 2 adverse events were hand-foot syndrome 57 cases (68.7%), diarrhea 45 cases (54.2%), alopecia 43 cases (51.8%), fatigue 41 cases (49.3%), anepithymia 39 cases (47.0%), nausea and vomiting 39 cases (47.0%), weight loss 38 cases (45.8%), and rash 38 cases (45.8%) (Table 2). Patients with grade 1 or 2 adverse events did not undergo sorafenib suspension or dose reduction after symptomatic treatment of the adverse events. Grade 3 or 4 adverse events included 5 cases (6%) of hand-foot syndrome, 4 cases (4.8%) of hypertension, and 3 cases (3.6%) of diarrhea. After dose reduction or suspension, patients who had grade 3 or 4 adverse events returned to grade 1 or 2. No patients had recurrence of grade 3 or 4 adverse events after that.

**TABLE 2:**
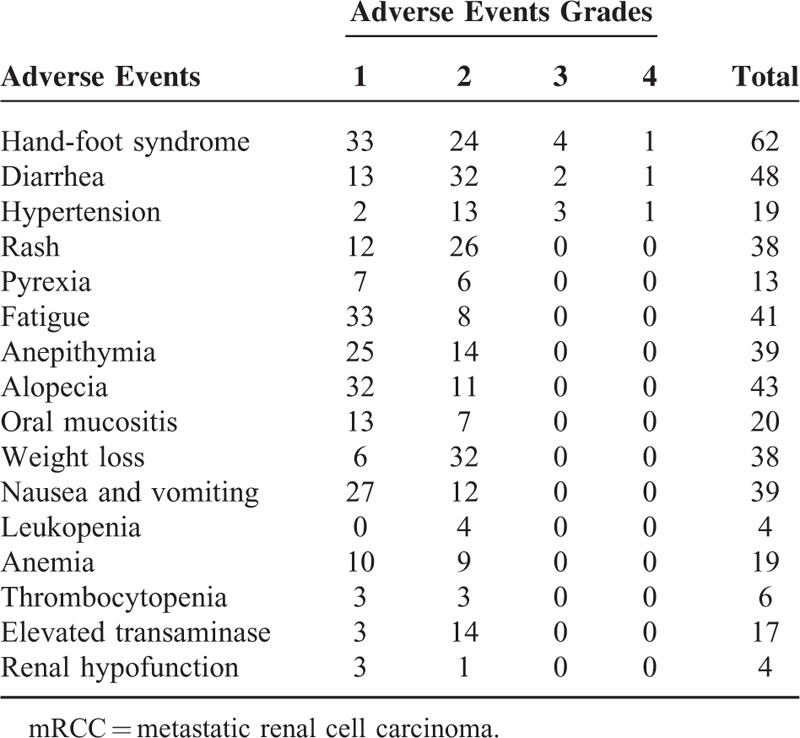
Adverse Events in Patients With mRCC Treated With Sorafenib

According to the types of adverse events, the patients were divided into 3 groups: 1 to 3, 4 to 6, and 7 or more types of adverse events. There was a positive correlation between the number of adverse event types and tumor response (χ^2^ = 19.358, *P* = 0.004). The more the types of adverse events were, the higher the tumor control rate was. Both patients with CR belonged to the group with 7 or more types of adverse events. Among the 10 cases of PD, 7 of them had ≤3 types of adverse events (Table 3). In addition, according to the highest levels of the adverse events, the patients were classified into 3 groups: grade 1, grade 2, and grade 3 or 4. The tumor response rates in patients with different grades of adverse events were also different (χ^2^ = 18.361, *P* = 0.005) and there were no adverse events higher than grade 1 in all 10 of patients with PD (Table 4).

**TABLE 3:**
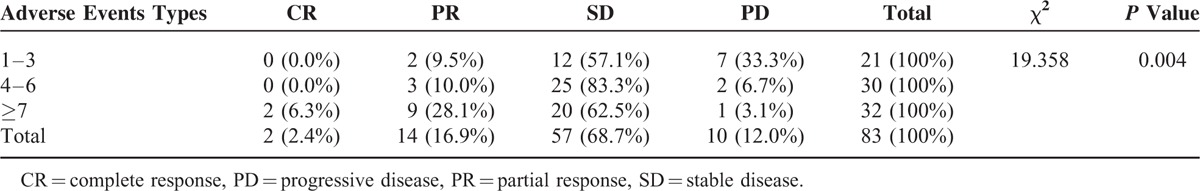
Comparison of Tumor Response Rate Among Different Adverse Events Types

**TABLE 4:**
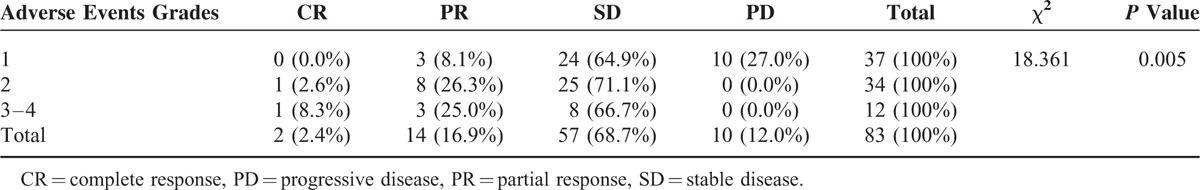
Comparison of Tumor Response Rate Among Different Adverse Events Grades

### Univariate Analysis

The data described above suggested that the numbers and the severity of adverse events positively correlated with tumor response. We next performed univariate analysis to determine what adverse events were related to tumor response. The following adverse events were associated with PFS: diarrhea, rash, fatigue, alopecia, weight loss, anemia, thrombocytopenia, and elevated transaminase (*P* < 0.05 for all these adverse events). The adverse events associated with OS included diarrhea, rash, fatigue, nausea and vomiting, and anemia (*P* < 0.05 for all these events, Table 5).

**TABLE 5:**
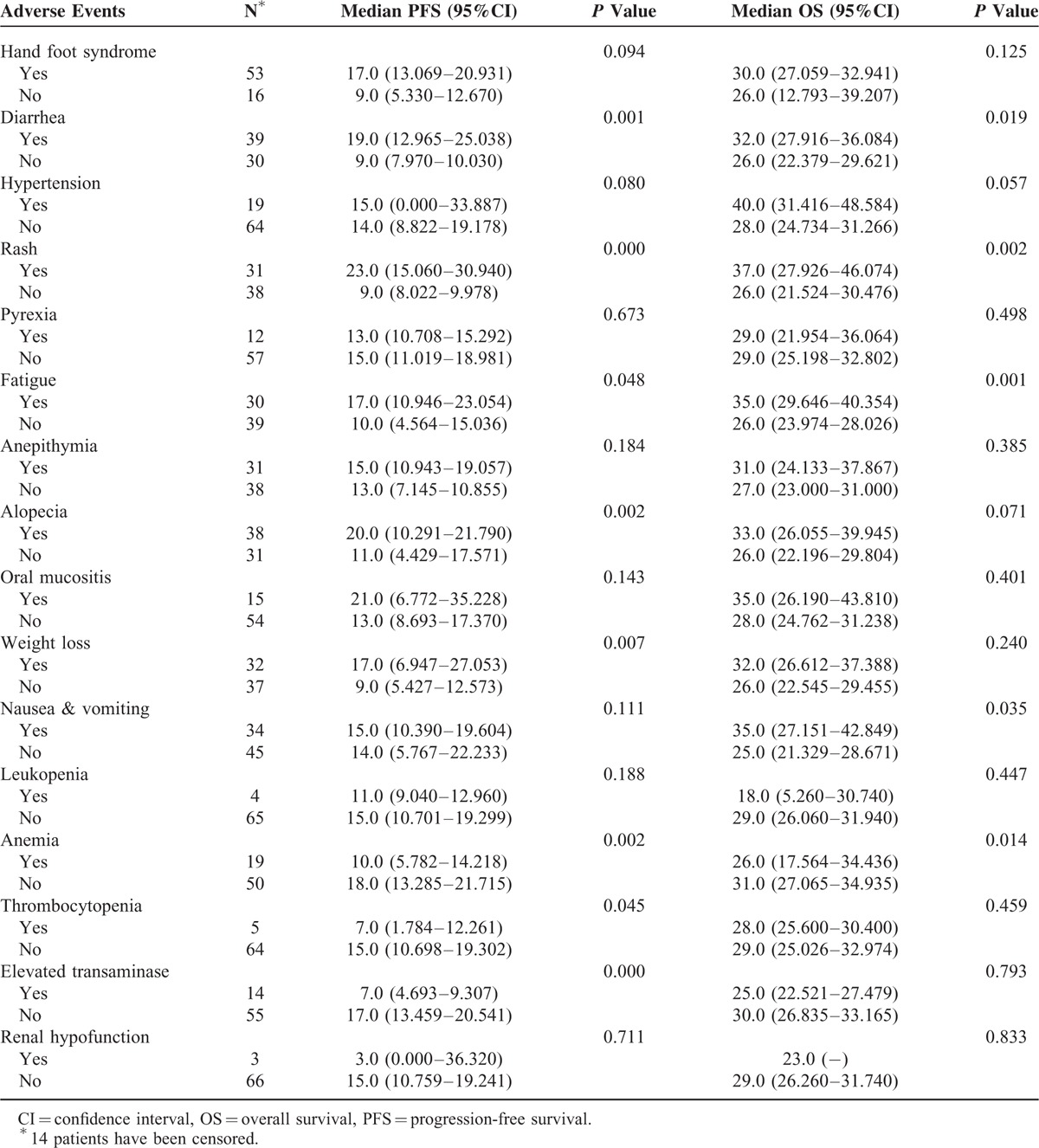
The Log-Rank Test Univariate Analysis for Progression-Free Survival and Overall Survival of mRCC Patients Treated With Sorafenib

### Multivariate Analysis

**FIGURE 1:**
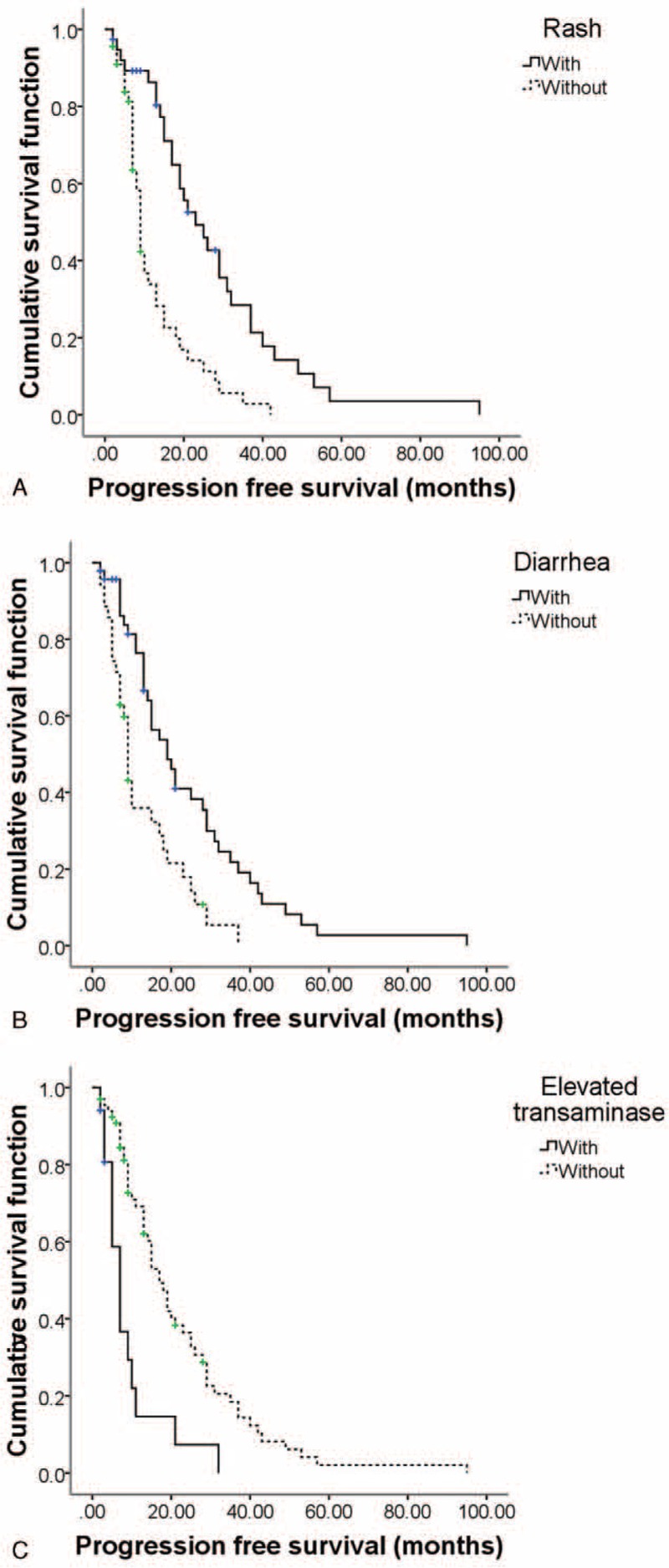
The Kaplan–Miller method cumulative PFS curves associated with adverse events: (A) rash, (B) diarrhea, (C) elevated transaminase. PFS = progression-free survival.

**FIGURE 2:**
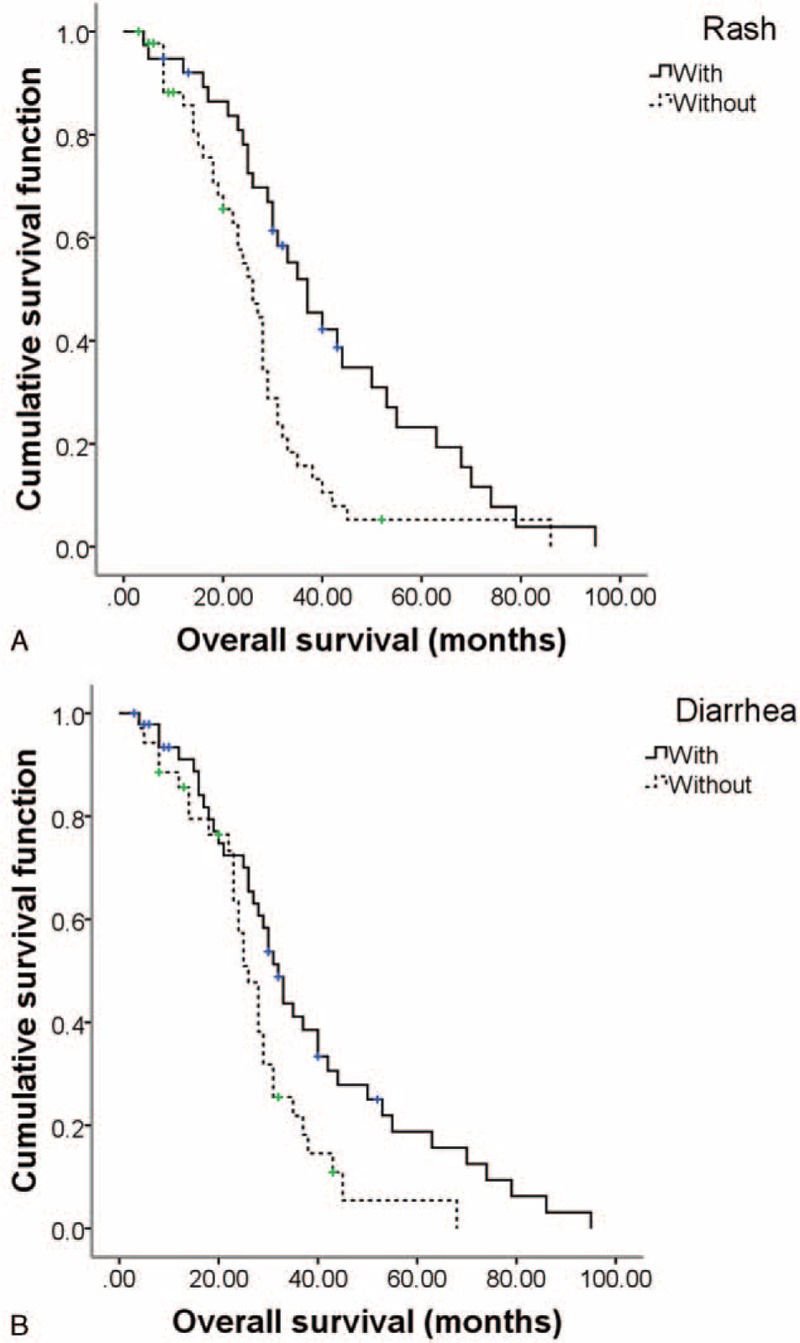
The Kaplan–Miller method cumulative OS curves associated with adverse events: (A) rash, (B) diarrhea. OS = overall survival.

Multivariable Cox proportional hazards model was used to identify adverse events, which are significantly associated with PFS or OS. In order to address potential sources of bias, 23 clinical and pathological variables were included in univariate analysis too. The positive results were also subjected to multivariate analysis. Results are shown in Supplementary Table S1 and Table S2. Rash and diarrhea were both independent predictive factors of better median PFS after sorafenib treatment with the odds ratio (OR) of 0.307 (95%CI 0.148–0.636, *P* = 0.001) and 0.391 (95%CI 0.169–0.783, *P* = 0.008), respectively (Table 6). However, elevated transaminase was the independent predictor of worse PFS with the OR of 2.606 (95%CI 1.299–5.532, *P* = 0.012). The Kaplan–Meier survival curves are showed in Figure 1. For OS, the independent predictive factors included rash (OR 0.473, 95%CI 0.253–0.886, *P* = 0.019) and diarrhea (OR 0.321, 95%CI 0.171–0.605, *P* = 0.000, Table 6). The Kaplan–Meier survival curves are showed in Figure 2.

**TABLE 6:**
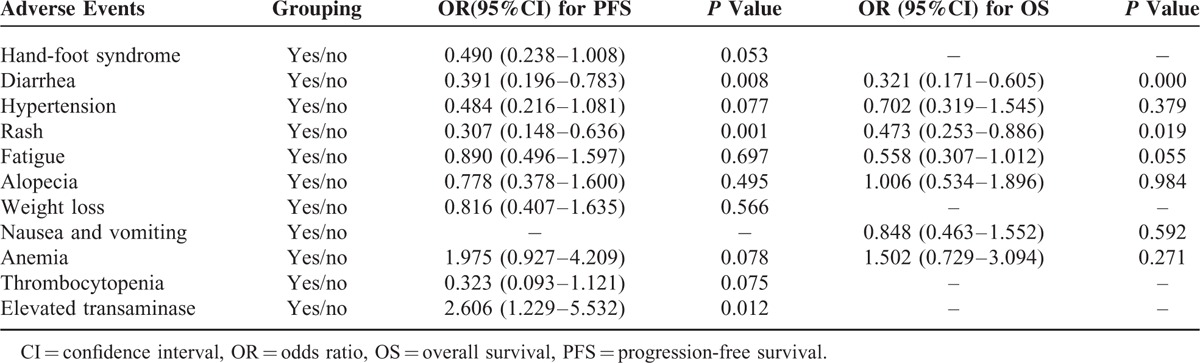
The Cox Proportional Hazards Multivariate Analysis for Progression Free Survival and Overall Survival of mRCC Patients Treated With Sorafenib

## DISCUSSION

Although targeted therapies for mRCC have been widely used in the world, many patients do not benefit from it and this treatment is still expensive. Therefore screening out the factors associated with efficacy to guide clinical use of targeted drugs is particularly important. A study has reported that some of the factors including pathological classification, sarcomatoid differentiation, and disease progression are associated with long-term survival of mRCC patients receiving targeted therapy.^6^ However, ideal predictive factors should be available before treatment which were easily obtained and inexpensive. Although adverse events are the predictive factors that occur after treatment starts, they are easily obtained and inexpensive. This multicenter retrospective study from Northwest China was aimed to identify predictive factors of the adverse events associated with PFS and OS to sorafenib therapy in mRCC.

Our data suggests that the severity and the number of the types of adverse events positively associated with the response of sorafenib treatment. The multivariate analysis suggested that different adverse events had different predictive values. Rash indicated better PFS and OS in mRCC patients treated with sorafenib, the OR values were 0.307 (95%CI 0.148–0.636, *P* = 0.001) and 0.473 (95%CI 0.253–0.886, *P* = 0.019), respectively. These results are similar to Poprach et al^10^ in which they reported an association of borderline significance between improved PFS and skin toxicity during sorafenib treatment, and the presence of cutaneous toxicity was related to improved OS and PFS in sunitinib therapy. As for other targeted therapies using TKIs in different kinds of tumors, an association of dermatologic toxicity with better treatment outcomes was commonly observed.^7–10,15–18^ It is not fully understood why the occurrence of the skin lesions could improve the efficacy. Because sorafenib is an inhibitor of the epidermal growth factor receptor (EGFR) that is highly expressed in the tumor tissue as well as in normal tissues, especially in the base layer of the epidermis,^19^ the expression of EGFR and dose-dependent toxicity at least partly explain the association of skin toxicity with treatment outcomes. There are several factors that could affect the development of skin toxicity as well. Tsuchiya et al^20^ suggested that female, high-dose sorafenib therapy (include dose per body weight and dose per body surface area), and ABCC-24CC or HLA-A^∗^24 carriers, are more prone to high-grade treatment-related rash.

Diarrhea is also an independent protective factor for both PFS and OS in mRCC patients treated with sorafenib; the OR values were 0.391 (95%CI 0.169–0.783, *P* = 0.008) and 0.321 (95%CI 0.171–0.605, *P* = 0.000), respectively. Strumberg et al^21^ found that patients who developed diarrhea during sorafenib treatment in phase II/III trials had a significantly longer time to progression compared to those without diarrhea. Koschny et al^22^ reported that during sorafenib therapy in patients with hepatocellular carcinoma, occurrence of grade 2 or 3 diarrhea was a good prognostic factor for OS. It is possible that the mechanism of skin toxicity and diarrhea is similar because gastrointestinal epithelium also highly expresses EGFR.^23^ As the diarrhea occurs less frequently with EGFR monoclonal antibodies treatment,^24^ some diarrhea may be related to the gastrointestinal tract irritation induced by the drug itself, it is the inflammatory reaction of the gut. These kinds of diarrhea could be alleviated by glucocorticoid,^25^ but the diarrhea caused by EGFR blockage could not. Hence, glucocorticoid therapy may become a marker for screening diarrhea as a prognostic factor.

Although the adverse events may not be fatal, they can lower the quality of life and make patients less compliance with the therapy and decrease efficacy. As these adverse events are predictive factors, it is important to explain to patients that dermatologic toxicity and diarrhea is associated with improved efficacy of the targeted therapy, at the same time, aggressive symptomatic treatment of adverse events could alleviate the toxicity, improve compliance, and enhance the efficacy.

Some patients are less likely to develop these toxicity as a result of the interaction between the pharmacokinetics^26^ and the genetic polymorphisms.^27,28^ They need higher dosage to induce the development of adverse events associated with efficacy. However, increasing the dosage of the drug is undoubtedly a double-edged sword because it can elevate transaminase that is an independent risk factor to worse PFS; the OR value is 2.606 (95%CI 1.299–5.532, *P* = 0.012). The elevated transaminase in high-dose sorafenib treatment may be simply because sorafenib is mainly metabolized in the hepatocyte.^29^

The adverse events can be divided into 3 categories: those associated with improved efficacy, those not related to efficacy, and those predicting worse treatment outcomes. For the adverse events associated with improved efficacy which include rash and diarrhea, aggressive supportive care should be taken to mitigate the toxicity, increase the compliance, and minimize the dose reduction or treatment interruption. As regards the adverse events not related to or associated with worse treatment outcomes, lower threshold should be set for dose reduction or drug holiday in addition to aggressive supportive care.

There are shortcomings of this study. This is the first such report focusing on Asian mRCC patients treated with sorefenib. All patients in this study were from the northwest of China. However, it is not clear whether the findings in this study can be applied to other Asian populations. With its relatively small size of 83 patients, some association between adverse events and treatment outcomes might not be found. There are many other prognostic factors affecting survival, such as primary tumor resection, Fuhrman grade, and so on (Supplementary Table S2). Unfortunately, due to the limited sample size, we could not conduct a subgroup analysis. With the continued expansion of the sample size, we will further strengthen this part in future studies.

## CONCLUSION

In conclusion, this study shows that some drug-related adverse events are associated with efficacy in mRCC treated with sorafenib. Rash and diarrhea are independent protective factors of both PFS and OS, whereas elevated transaminase is an independent risk factor of PFS. These findings remain to be validated by large prospective studies.

## Supplementary Material

Supplemental Digital Content
