# Supplementary material for: The Relationship Between the Adverse Events and Efficacy of Sorafenib in Patients With Metastatic Renal Cell Carcinoma: A Multicenter Retrospective Study from Northwest China
Source: Medicine (Baltimore). 2015 Dec 11;94(49):e2222. doi: 10.1097/MD.0000000000002222 (PMC5008507; doi:10.1097/MD.0000000000002222)
Supplement: Supplemental Digital Content [file medi-94-e2222-s001.docx]

**SUPPLEMENTARY TABLE S1.** The Log-rank test univariate analysis of clinical and pathological variables for progression free survival and overall survival of mRCC patients treated with sorafenib

| Variables | *N*^*^ | Median PFS（95%CI） | *P* value | Median OS（95%CI） | *P* value |
| --- | --- | --- | --- | --- | --- |
| Gender  Male  Female  Age  ≤60  ≥61  Ethnic group  Han  Others  Smoking  Yes  No  Drinking  Yes  No  Hypertension  Yes  No  Cerebrovascular disease  Yes  No  Diabetes  Yes  No  Primary tumor resection  Yes  No  Focus anatomical site  Unilateral  Bilateral  Pathological classification  Clear cell carcinoma  Non-clear cell carcinoma  Pathological stage  ≤T2  ≥T3  Clinical stage  3  4  Funrmann grade  ≤2  ≥3  Venous invasion  Yes  No  Lymph node metastasis  Yes  No  Number of organ metastases  ≤1  ≥2  Lung metastasis  Yes  No  Liver metastases  Yes  No  Bone metastasis  Yes  No  Adrenal violations  Yes  No  ECOG score  0  ≥1  Time from diagnosis to treatment  ＜6 months  ≥6 months | 50  19  46  23  67  2  19  50  12  57  16  53  4  65  4  65  64  5  67  2  64  5  41  28  2  67  52  17  22  47  14  55  40  29  37  32  13  56  9  60  7  62  42  27  58  11 | 15.0(9.187-20.813)  14.0(10.011-17.989)  18.0(12.384-23.616)  13.0(8.912-17.088）  15.0(11.542-18.458)  29.0(—)  17.0(3.665-30.335)  15.0(12.015-17.985)  26.0(0.000-55.767)  15.0(11.830-18.160)  15.0(10.109-19.891)  13.0(8.002-17.998)  35.0(0.000-79.909)  15.0(10.787-19.213)  15.0(3.024-24.976)  15.0(9.812-20.188)  15.0(9.639-10.361)  11.0(0.000-15.602)  14.0(10.548-17.452)  32.0(—)  15.0(10.661-19.339)  10.0(4.280-15.720)  19.0(13.657-24.343)  10.0(5.991-14.009)  17.0(—)  15.0(11.538-18.462)  17.0(13.169-20.832)  9.0(4.515-13.485)  15.0(8.378-21.622)  15.0(10.640-19.360)  11.0(4.765-17.254)  15.0(9.953-20.047)  21.0(12.725-29.275)  11.0(8.462-13.538)  13.0(9.186-16.814)  18.0(11.548-24.452)  13.0(2.000-24.000)  15.0(11.516-18.484)  15.0(5.952-25.048)  15.0(10.165-19.835)  9.0(4.080-13.920)  15.0(10.915-19.085)  20.0(9.904-30.096)  9.0(6.579-11.421)  15.0(9.510-20.490)  10.0(6.625-13.375) | 0.463  0.400  0.222  0.869  0.315  0.879  0.391  0.412  0.037  0.218  0.112  0.015  0.595  0.000  0.628  0.363  0.001  0.571  0.464  0.892  0.608  0.000  0.079 | 28.0(23.643-32.357)  31.0(25.388-36.612)  29.0(25.167-32.883)  28.0(22.990-33.010)  28.0(25.224-30.776)  35.0( — )  29.0(19.101-38.899)  29.0(26.285-31.715)  31.0(15.812-46.188)  29.0(26.462-31.538)  30.0(24.244-35.756)  28.0(24.063-31.935)  35.0(22.118-47.882)  28.0(24.398-31.602)  28.0(19.412-36.588)  29.0(25.963-32.037)  30.0(27.313-32.687)  12.0(4.160-19.840)  28.0(24.921-31.079)  40.0( — )  29.0(25.359-32.641)  27.0(12.501-41.499)  31.0(26.694-35.306)  26.0(23.373-28.627)  79.0( — )  28.0(24.921-31.079)  32.0(27.318-36.682)  24.0(19.220-28.780)  27.0(22.345-31.655)  29.0(25.515-32.485)  28.0(23.202-32.798)  30.0(26.222-33.778)  37.0(30.813-43.187)  21.0(15.806-26.194)  26.0(22.976-29.024)  31.0(26.537-35.463)  22.0(11.000-33.000)  30.0(26.799-33.201)  23.0(14.368-31.632)  30.0(27.211-32.789)  24.0(15.684-32.316)  29.0(26.362-31.638)  37.0(30.687-43.313)  23.0(20.548-25.452)  28.0(24.307-31.693)  45.0(20.746-69.254) | 0.460  0.837  0.593  0.240  0.571  0.320  0.480  0.945  0.000  0.204  0.261  0.183  0.074  0.001  0.852  0.052  0.000  0.194  0.004  0.409  0.418  0.000  0.007 |

ECOG = Eastern Cooperative Oncology Group, OS = overall survival, PFS = progression free survival.

^*^14 patients have been censored.

**SUPPLEMENTARY TABLE S2.** The Cox proportional hazards multivariate analysis of all positive variables for progression free survival and overall survival of mRCC patients treated with sorafenib

| Variables | Grouping | OR（95%CI）for PFS | *P* value | OR（95%CI）for OS | *P* value |
| --- | --- | --- | --- | --- | --- |
| Primary tumor resection  Pathological stage  Funrmann grade  Lymph node metastasis  Number of organ metastases  Liver metastases  ECOG score  Time from diagnosis to treatment  Hand-foot syndrome  Diarrhea  Hypertension  Rash  Fatigue  Alopecia  Weight loss  Nausea & vomiting  Anemia  Thrombocytopenia  Elevated transaminase | Yes/No  ≥T3/≤T2  ≥3/≤2  Yes/No  ≥2/≤1  Yes/No  ≥1/0  ≥6 months/＜6 months  Yes/no  Yes/no  Yes/no  Yes/no  Yes/no  Yes/no  Yes/no  Yes/no  Yes/no  Yes/no  Yes/no | 0.180(0.053-0.608)  0.625(0.336-1.161)  3.709(1.625-8.463)  —  1.813(0.971-3.387)  —  1.433(0.679-3.022)  5.513(2.304-13.192)  0.490(0.238-1.008)  0.391(0.196-0.783)  0.484(0.216-1.081)  0.307(0.148-0.636)  0.890(0.496-1.597)  0.778(0.378-1.600)  0.816(0.407-1.635)  ­­—  1.975(0.927-4.209)  0.323(0.093-1.121)  2.606(1.229-5.532) | 0.006  0.137  0.002  —  0.062  —  0.345  0.000  0.053  0.008  0.077  0.001  0.697  0.495  0.566  —  0.078  0.075  0.012 | 0.103(0.030-0.357)  —  3.074(1.410-6.702)  2.347(1.075-5.124)  2.311(1.202-4.442)  3.007(1.359-6.654)  1.902(0.808-4.480)  0.532(0.244-1.158)  —  0.321(0.171-0.605)  0.702(0.319-1.545)  0.473(0.253-0.886）  0.558(0.307-1.012)  1.006(0.534-1.896)  —  0.848(0.463-1.552)  1.502(0.729-3.094)  —  — | 0.000  —  0.005  0.032  0.012  0.007  0.141  0.112  —  0.000  0.379  0.019  0.055  0.984  —  0.592  0.271  —  — |

CI = confidence interval, OR = odds ratio, OS = overall survival, PFS = progression free survival.
